# White Blood Cell Count Is Associated With Hospitalisation for Heart Failure in Patients With ATTR Cardiac Amyloidosis

**DOI:** 10.1002/clc.70525

**Published:** 2026-11-10

**Authors:** Elias Haj‐Yehia, Julia Vogel, Samuel Dautzenberg, Sophia Jura, Phillip Schulte, Stephan Settelmeier, Florian Buehning, Tobias Lerchner, Loubna Yahsaly, Alexander Carpinteiro, Christian Reinhardt, Matthias Totzeck, Tienush Rassaf, Lars Michel

**Affiliations:** ^1^ Department of Cardiology and Vascular Medicine West German Heart and Vascular Center, University Hospital Essen Essen Germany; ^2^ West German Amyloidosis Center University Hospital Essen Essen Germany; ^3^ Department of Hematology and Stem Cell Transplantation West German Cancer Center, University Hospital Essen Essen Germany; ^4^ DKTK‐German Cancer Consortium Site Essen‐Düsseldorf Essen Germany

**Keywords:** ATTR, cardiac amyloidosis, inflammation, neutrophils, transthyretin amyloid cardiomyopathy, white blood cell count

## Abstract

**Aims and Background:**

Transthyretin amyloid cardiomyopathy (ATTR‐CM) is the most common form of cardiac amyloidosis leading to restrictive cardiomyopathy and progressive heart failure. The prognostic relevance of inflammatory biomarkers is unknown so far. This study aims to evaluate an association of white blood cell (WBC) count and clinical outcome in form of hospitalisation for heart failure in ATTR‐CM patients.

**Methods and Results:**

WBC count was assessed in 150 patients (median age 80 [76−82] years; 13.3% female) with ATTR‐CM from the West German Amyloidosis Center. Most patients (95.3%) received transthyretin stabilizer therapy. The cohort was divided into patients with low and high WBC count by median split. Hospitalisation for heart failure was defined as primary endpoint. The median follow‐up time was 825 (728−932) days. ATTR‐CM patients with high WBC count showed higher prevalence of heart failure with reduced ejection fraction (HFrEF) at baseline assessment. High WBC count was mainly driven by a relative increase in neutrophils. During follow‐up incidence of hospitalisation for heart failure was higher in patients with high WBC count (log‐rank *p* = 0.035). Cox regression confirmed an association between WBC count and occurrence of hospitalisation due to heart failure (hazard ratio [HR]: 1.130; 95% confidence interval [CI]: 1.015−1.257; *p* = 0.025), which was unaffected after adjustment for further confounders.

**Conclusions:**

Increased WBC count was associated with incidence of hospitalisation for heart failure in ATTR‐CM patients, indicating a potential for inflammatory biomarkers in disease monitoring.

## Graphical abstract

Increased white blood cell (WBC) count was associated with incidence of heart failure hospitalisation in patients with transthyretin amyloid cardiomyopathy (ATTR‐CM), indicating a potential for inflammatory biomarkers in disease monitoring.

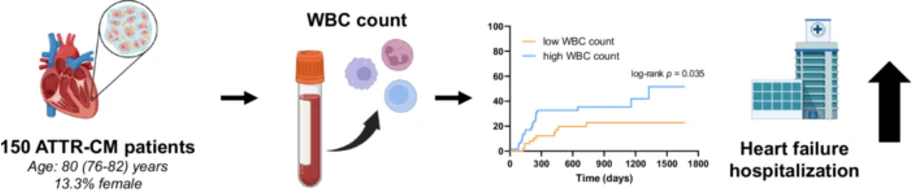

## 1 Introduction

Cardiac amyloidosis is characterized by abnormal amyloid protein deposition in the heart, leading to restrictive cardiomyopathy with impairment of cardiac function and heart failure [1]. The most common form of cardiac amyloidosis is transthyretin amyloid cardiomyopathy (ATTR‐CM), which primarily affects elderly individuals [2]. ATTR‐CM is induced by cardiac extracellular deposits of wild‐type or variant transthyretin (TTR) monomers with subsequent formation of amyloid fibrils [3, 4]. Recently, increased awareness and improved diagnosis have led to a profound increase in reported incidence of ATTR‐CM [5]. Specific disease‐modifying drugs are available, including transthyretin stabilizers and silencers [6, 7, 8, 9, 10]. However, prognosis of patients with ATTR‐CM is still limited [11]. With new therapeutic alternatives in development, valid biomarkers for monitoring of disease progression and therapy response are essential, as this remains challenging in clinical practice so far [12, 13, 14].

For different forms of cardiovascular diseases, including coronary artery disease, acute myocardial infarction, and common heart failure, inflammation is involved in disease development and progression—thus also leading to the identification of promising novel inflammation‐related biomarkers [15, 16, 17]. Although ATTR‐CM has long been considered a non‐inflammatory cardiomyopathy, recent evidence suggests a potential role of inflammation in its pathogenesis and progression [18]. Observations from endomyocardial biopsy show myocardial inflammation in one third of ATTR‐CM patients [19]. Levels of different inflammatory cytokines can be elevated in patients with cardiac amyloidosis, but there is no available evidence on its role as a potential biomarker for disease progression [20]. This study aims to analyse the association of white blood cell (WBC) count and the risk for hospitalisation due to heart failure in ATTR‐CM patients.

## 2 Methods

### 2.1 Study Population

For this retrospective analysis, we screened patients (≥18 years) diagnosed with ATTR‐CM at the West German Amyloidosis Center of the University Hospital Essen between January 2018 and December 2023, who provided written informed consent. This study was approved by the ethics committee of the University Duisburg‐Essen (23‐11500‐BO). Patients without a differential blood count were excluded from further analysis. Diagnosis of ATTR‐CM was established according to current European guidelines, based on 99mTc‐DPD scintigraphy in combination with clinical findings. In cases of diagnostic uncertainty or atypical findings, endomyocardial biopsy was performed. Genetic testing was used to differentiate between wild‐type (ATTRwt) and hereditary (ATTRv) transthyretin amyloidosis [1]. Staging of ATTR‐CM was performed according to National Amyloidosis Centre (NAC) ATTR stages [21]. Data of baseline demographics, co‐morbidities, cardiovascular medication (including initiation of disease‐modifying therapy), as well as echocardiographic, hemodynamic, and laboratory parameters were systematically gathered from the clinic's internal data system. Echocardiographic measurements included left‐ventricular ejection fraction (LV‐EF), left ventricular wall thickness (IVSD), diastolic function and stroke volume (SV), among others. In addition to routine laboratory values, C‐reactive protein (CRP) levels were measured at baseline as part of the inflammatory profile. The primary endpoint was defined as hospitalisation for heart failure. N‐terminal pro b‐type natriuretic peptide (NT‐proBNP), estimated glomerular filtration rate (eGFR), systolic blood pressure (SBP), and WBC count were recorded after 6 and 12 months of patient follow‐up.

### 2.2 Statistical Analysis

Categorical variables are presented as absolute and relative frequencies (%). Association between these variables was examined by chi‐squared test. Continuous variables were expressed as median with IQR and compared using the Mann–Whitney *U* test. In case of multiple comparisons for continuous variables, two‐way ANOVA with Šidák post hoc test was used. Kaplan‐Meier cumulative event curves displayed the rate of hospitalisation for heart failure in dependence of low and high WBC count and were compared using the log‐rank test. Patients lost to follow‐up were treated as censored observations. Median follow‐up time was calculated by the reverse Kaplan–Meier method. The association between WBC count and hospitalisation for heart failure was examined using univariable Cox regression. Multivariable Cox regression models were used to adjust for relevant clinical covariates and confirm the independent prognostic value of WBC count. Adjustments covered cardiovascular comorbidities and risk factors, namely coronary artery disease, valvular disease, heart failure, hypertension, dyslipidemia, diabetes, and obesity. In addition, heart failure therapies with potential impact on hospitalization risk were considered, including β‐blockers, renin–angiotensin–aldosterone system (RAAS) inhibitors, and sodium–glucose cotransporter‐2 (SGLT‐2) inhibitors. The significance level was set as *p* < 0.05. All statistical analyses were performed using IBM SPSS 29.0 software (SPSS Inc.). Figures were generated with GraphPad Prism 9.5.1 software (GraphPad Software Inc.). Elements of the graphical abstract were created with BioRender. com.

## 3 Results

After screening of 240 consecutive patients, 150 ATTR‐CM patients (median age 80 years [IQR: 76−82 years]; 13.3% female; Table 1) with examined differential blood counts were included for further analysis (Figure 1). The final study population was divided by median split of WBC count into two groups of patients with low (≤ 7.36 cells/nL) and high (> 7.36 cells/nL) WBC count (Figure 1). Clinical baseline characteristics of the study collective according to low and high WBC count are depicted in Table 1, while information on baseline medication is shown in Table 2.

**Table 1:** Baseline characteristics.

	Total (*n* = 150)	Low WBC count (*n* = 75)	High WBC count (*n* = 75)	*p* value
Age (years), median (IQR)	80 (76‐82)	80 (76‐83)	79 (73‐82)	0.09
Sex (female)	20 (13.3%)	10 (13.3%)	10 (13.3%)	1.00
ATTR‐CM diagnosis				
Scintigraphy	112 (74.7%)	59 (78.7%)	53 (70.7%)	0.26
Perugini grade < 3	33 (22.0%)	18 (24.0%)	15 (20.0%)	0.80
Perugini grade ≥ 3	79 (52.7%)	41 (54.7%)	38 (50.7%)	0.80
Endomyocardial biopsy	87 (58.0%)	38 (50.7%)	49 (65.3%)	0.07
Hereditary ATTR‐CM (ATTRv)	7 (4.7%)	2 (2.7%)	5 (6.7%)	0.24
ATTR‐CM staging				
NAC < 2	59 (39.3%)	30 (40.0%)	29 (38.7%)	0.87
NAC ≥ 2	91 (60.7%)	45 (60.0%)	46 (61.3%)	0.87
NYHA class				
II	88 (58.7%)	52 (69.3%)	36 (48.0%)	0.008*
III	53 (36.0%)	17 (22.7%)	37 (49.3%)	< 0.001*
IV	8 (5.3%)	6 (8.0%)	2 (2.7%)	0.15
Comorbidities				
Hypertension	99 (66.0%)	49 (65.3%)	50 (66.7%)	0.86
Dyslipidemia	68 (45.3%)	33 (44.0%)	35 (46.7%)	0.74
Diabetes mellitus	35 (23.3%)	16 (21.3%)	19 (25.3%)	0.56
History of smoking (current or past)	50 (33.3%)	22 (29.3%)	28 (37.3%)	0.30
BMI (kg/m^2^), median (IQR)	25.5 (23.4‐28.0)	25.2 (23.0‐27.5)	26.1 (23.5‐29.1)	0.11
Obesity	17 (11.3%)	5 (6.7%)	12 (16.0%)	0.07
Coronary artery disease	73 (48.7%)	35 (46.7%)	38 (50.7%)	0.62
History of PCI	46 (30.7%)	24 (32.0%)	22 (29.3%)	0.72
CABG	9 (6.0%)	6 (8.0%)	3 (4.0%)	0.30
Peripheral artery disease	7 (4.7%)	4 (5.3%)	3 (4.0%)	0,70
HFpEF	91 (60.7%)	46 (61.3%)	45 (60.0%)	0.87
HFmrEF	31 (24.7%)	20 (26.7%)	1 (14.7%)	0.07
HFrEF	28 (18.7%)	9 (12.0%)	19 (25.3%)	0.04*
Aortic valve stenosis	17 (11.3%)	8 (10.7%)	9 (12.0%)	0.82
Mitral valve regurgitation	144 (96.0%)	74 (98.7%)	70 (93.3%)	0.02*
Mild	74 (49.3%)	38 (50.7%)	36 (48.0%)	0.68
Moderate	51 (34.0%)	30 (40.0%)	21 (28.0%)	0.11
Severe	19 (12.7%)	6 (8.0%)	13 (17.3%)	0.09
Tricuspid valve regurgitation	135 (90.0%)	70 (93.3%)	65 (86.7%)	0.10
Mild	81 (54.0%)	45 (60.0%)	36 (48.0%)	0.12
Moderate	32 (21.3%)	17 (22.7%)	15 (20.0%)	0.66
Severe	22 (14.7%)	8 (10.7%)	14 (18.7%)	0.18
Pacemaker or ICD	23 (15.3%)	11 (14.7%)	12 (16.0%)	0.82
Atrial fibrillation	104 (69.3%)	52 (69.3%)	52 (69.3%)	1.00
History of stroke	18 (12.0%)	11 (14.7%)	7 (9.3%)	0.32
COPD	10 (6.7%)	4 (5.3%)	6 (8.0%)	0.62

*Note:* Continuous variables are expressed as median with interquartile range (IQR). Categorical variables are shown as frequencies and percentages (%).

Abbreviations: ATTR‐CM, transthyretin amyloid cardiomyopathy; BMI, body mass index; CABG, coronary artery bypass graft; COPD, chronic obstructive pulmonary disease; HFmrEF, heart failure with mildly reduced ejection fraction; HFpEF, heart failure with preserved ejection fraction; HFrEF, heart failure with reduced ejection fraction; ICD, implantable cardioverter‐defibrillator; NAC, National Amyloidosis Centre; NYHA, New York Heart Association; PCI, percutaneous coronary intervention; WBC, white blood cell.

* Statistically significant difference between patients with low and high WBC count.

**Figure 1:**
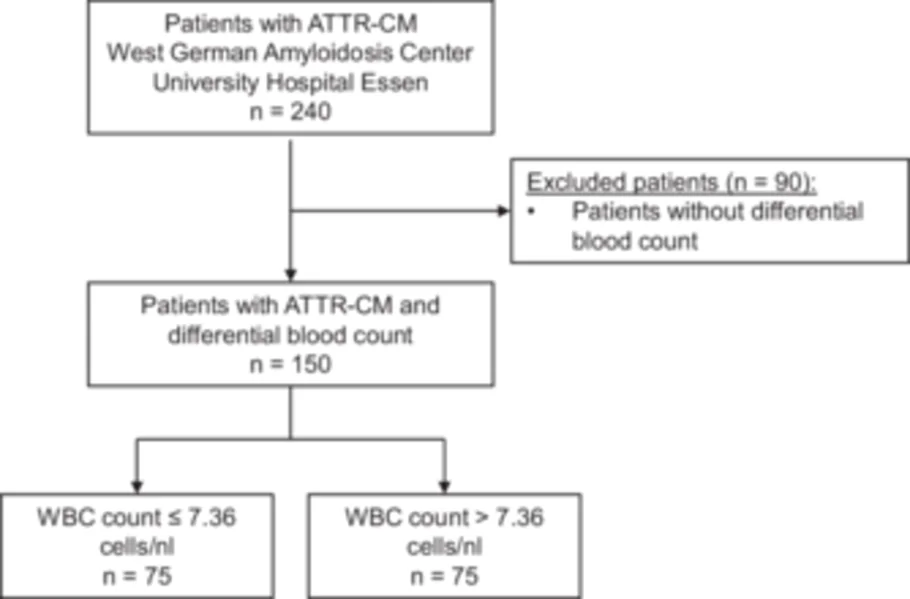
Flow chart of the study population. Out of 240 patients diagnosed with transthyretin amyloid cardiomyopathy (ATTR‐CM) from the West German Amyloidosis Center, 150 patients with differential blood counts were identified. The study population was further divided into patients with low (≤ 7.36 cells/nl) and high (> 7.36 cells/nl) white blood cell (WBC) count by median split.

**Table 2:** Baseline medication.

	Total (*n* = 150)	Low WBC count (*n* = 75)	High WBC count (*n* = 75)	*p* value
β blocker	86 (57.3%)	44 (58.7%)	42 (56.0%)	0.81
ACEi or ARB	75 (50.0%)	39 (52.0%)	36 (48.0%)	0.68
MRA	64 (42.7%)	32 (42.7%)	32 (42.7%)	1.00
ARNI	7 (4.7%)	4 (5.3%)	3 (4.0%)	0.71
SGLT‐2 inhibitor	32 (42.7%)	17 (22.7%)	15 (20.0%)	0.72
Diuretics	110 (73.3%)	49 (65.3%)	61 (81.3%)	0.02*
Anticoagulation	104 (69.3%)	52 (69.3%)	52 (69.3%)	1.00
Antiplatelet therapy	44 (29.3%)	22 (29.3%)	22 (29.3%)	1.00
Statins	90 (60.0%)	45 (60.0%)	45 (60.0%)	1.00
Disease‐modifying therapy	143 (95.3%)	71 (94.7%)	72 (96.0%)	0.70

*Note:* Variables are shown as frequencies and percentages (%).

Abbreviations: ACEi, angiotensin‐converting enzyme inhibitor; ARB, angiotensin receptor blocker; ARNI, angiotensin receptor/neprilysin inhibitor; MRA, mineralocorticoid receptor antagonist; SGLT‐2, sodium‐dependent glucose co‐transporter 2; WBC, white blood cell.

* Statistically significant difference between patients with low and high WBC count.

Analysed patients showed similar NAC ATTR stages, but patients with high WBC count presented with more severe symptoms as a higher portion was in NYHA class III (17 patients [22.7%] vs. 37 patients [49.3%]; *p* < 0.001; Table 1). ATTRv amyloidosis with a predominant cardiomyopathy phenotype was found in seven patients (Table 1, Supporting Information S1: Table 1). ATTR‐CM patients with high WBC count also showed a higher prevalence of heart failure with reduced ejection fraction (HFrEF) compared to patients with low WBC (9 patients [12.0%] vs. 19 patients [25.3%]; *p* = 0.04; Table 1). In contrast, prevalence of any grade mitral valve regurgitation was slightly higher in patients with low WBC count (74 patients [98.7%] vs. 70 patients [93.3%]; *p* = 0.02; Table 1). In line with increased rates of HFrEF, the use of diuretics was more frequent in patients with high WBC count (49 patients [65.3%] vs. 61 patients [81.3%]; *p* = 0.02; Table 2). Besides, both groups showed similar rates of disease‐modifying therapy with tafamidis for ATTR‐CM (71 patients [94.7%] vs. 72 patients [96.0%]; *p* = 0.70; Table 2). Hemodynamic and echocardiographic measurements are depicted in Table 3, showing no significant differences between patients with low and high WBC count for assessment of SBP, heart rate, LV‐EF, IVSD, and diastolic function.

**Table 3:** Baseline hemodynamic and echocardiography parameters.

	Total (*n* = 150)	Low WBC count (*n* = 75)	High WBC count (*n* = 75)	*p* value
Hemodynamic parameters				
Systolic blood pressure (mmHg)	130 (118‐147)	129 (120‐148)	132 (118‐145)	0.98
Heart rate (bpm)	75 (65‐87)	72 (62‐86)	77 (66‐87)	0.10
Echocardiography parameters				
LV‐EF (%)	51 (45‐55)	51 (45‐55)	51 (40‐55)	0.49
Stroke volume (mL)	53 (45‐63)	53 (47‐66)	51 (44‐60)	0.14
LVMM (g)	314 (238‐387)	343 (244‐405)	302 (236‐375)	0.13
IVSD (mm)	18.4 (14.8‐21.1)	18.5 (15.6‐21.7)	17.8 (14.5‐21.0)	0.36
E/e’ ratio	12 (10‐16)	12 (10‐15)	14 (10‐18)	0.89
TAPSE (mm)	16.7 (14.0‐20.6)	16.6 (12.7‐20.4)	17.1 (14.2‐20.7)	0.90

*Note:* Variables are expressed as median with interquartile range (IQR).

Abbreviations: IVSD, interventricular septum diastole; LV‐EF, left ventricular ejection fraction; LVMM, left ventricular myocardial mass; TAPSE, tricuspid annular plane systolic excursion; WBC, white blood cell.

We further analysed different immune cell subpopulations in ATTR‐CM patients with low and high WBC count. Patients with high WBC count showed a significantly increase in absolute count of neutrophils, lymphocytes and monocytes (Table 4). However, after normalization to WBC count, the percentage of neutrophils was significantly increased and percentage of lymphocytes was decreased in patients with high WBC count (Figure 2). Accordingly, neutrophil‐to‐lymphocyte ratio (NLR) was elevated in patients with high WBC count (Table 4). In this group, the absolute count of thrombocytes was also increased (Table 4). CRP levels were not significantly elevated in patients with higher WBC count, suggesting that the observed leukocytosis was not associated with a systemic inflammatory response as measured by CRP (Table 4).

**Table 4:** Baseline laboratory parameters.

	Total (*n* = 150)	Low WBC count (*n* = 75)	High WBC count (*n* = 75)	*p* value
WBC (count/nL)	7.36 (6.28‐8.72)	6.29 (4.95‐6.69)	8.72 (8.06‐9.88)	< 0.001*
Neutrophils (count/nL)	5.05 (3.96‐6.28)	3.98 (3.12‐4.71)	6.27 (5.66‐7.19)	< 0.001*
Lymphocytes (count/nL)	1.31 (1.00‐1.80)	1.21 (0.90‐1.60)	1.53 (1.13‐1.95)	0.002*
Monocytes (count/nL)	0.60 (0.49‐0.76)	0.52 (0.46‐0.63)	0.68 (0.58‐0.92)	< 0.001*
Thrombocytes (count/nL)	193 (155‐227)	181 (151‐210)	200 (166‐247)	0.006*
Hemoglobin (g/dL)	13.6 (12.3‐14.5)	13.4 (12.2‐14.5)	13.8 (12.4‐14.6)	0.34
CRP (mg/dL)	0.4 (0.4‐0.5)	0.4 (0.4‐0.5)	0.4 (0.4‐0.6)	0.11
NLR	3.8 (2.7‐5.1)	3.3 (2.3‐4.5)	4.2 (3.3‐6.9)	< 0.001*
LMR	153 (93‐191)	152 (89‐181)	170 (101‐206)	0.22
PLR	2.5 (1.6‐3.5)	2.7 (1.6‐3.6)	2.3 (1.6‐3.3)	0.40
PMR	317 (242‐431)	331 (223‐435)	311 (279‐411)	0.77
hsTrop (ng/L)	45 (26‐75)	45 (27‐76)	42 (24‐74)	0.55

*Note:* Variables are expressed as median with interquartile range (IQR).

Abbreviations: CRP, C‐reactive protein; hsTrop, high‐sensitive troponin; LMR, lymphocyte‐to‐monocyte ratio; NLR, neutrophil‐to‐lymphocyte ratio; PLR, platelet‐to‐lymphocyte ratio; PMR, platelet‐to‐monocyte ratio; WBC, white blood cell;

* Statistically significant difference between patients with low and high WBC count.

**Figure 2:**
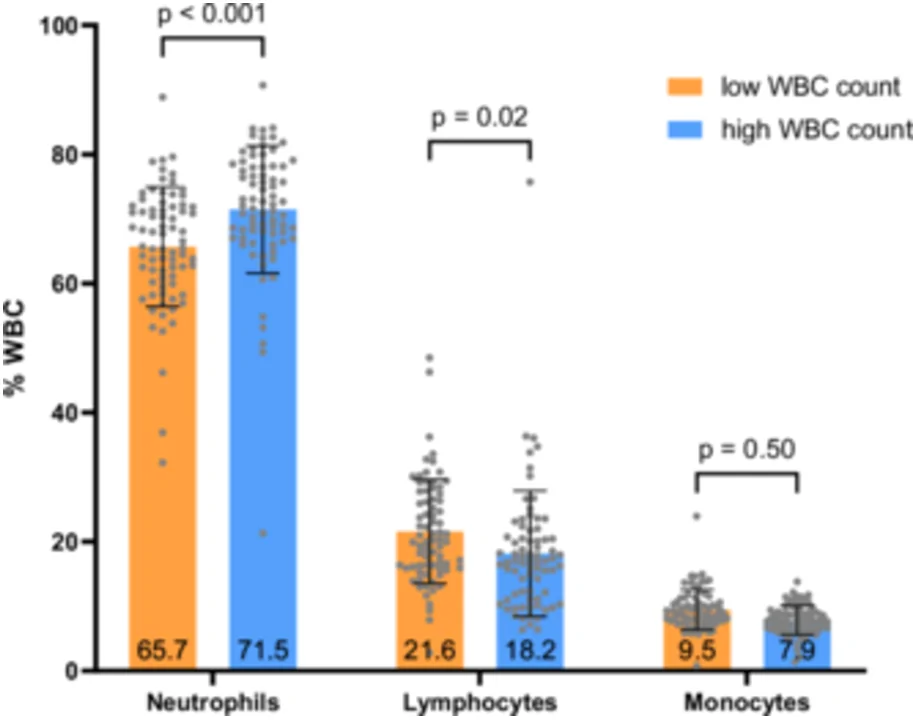
Baseline relative count of different immune cell subpopulations (neutrophils, lymphocytes and monocytes) normalized to total white blood cells (WBC) in patients with low and high WBC count. Numbers depicted in inside the columns represent the mean percentage of different immune cell subpopulations for each patient group.

The median follow‐up time was 825 days (interquartile range (IQR): 718−932 days). Unplanned hospitalisation for heart failure occurred in 37 patients (24.7%). Patients with high WBC count had significantly higher rates of heart failure‐related hospitalisation (log‐rank *p* = 0.035; Figure 3). Univariable Cox regression supported this observation, as patients with higher WBC count had an increased risk for heart failure hospitalisation (hazard ratio (HR): 1.130; 95% confidence interval (CI): 1.015−1.257; *p* = 0.025; Table 4). This association remained significant after adjustment for age and sex (HR: 1.134; 95% CI: 1.017−1.264; *p* = 0.023; Table 4 [Model 1]) or additional adjustment for other comorbidities (HR: 1.203; 95% CI: 1.028‐1.407; *p* = 0.021; Table 4 [Model 2]). This also applied to adjustment for baseline medication (HR: 1.204; 95% CI: 1.044−1.389; *p* = 0.011; Table 4 [Model 3]) or other clinical biomarkers as LV‐EF, SBP, hemoglobin, platelet count, CRP, eGFR, high‐sensitive troponin and NT‐proBNP (HR: 1.142; 95% CI: 1.008−1.295; *p* = 0.037; Table 4 [Model 4]). We also did not observe any significant differences of values for NT‐proBNP, eGFR, or SBP during follow‐up after 6 months or 12 months between patients with low and high WBC count (Figure 4). Baseline differences in WBC count persisted at 6 months and 12 months follow‐up (Figure 5).

**Figure 3:**
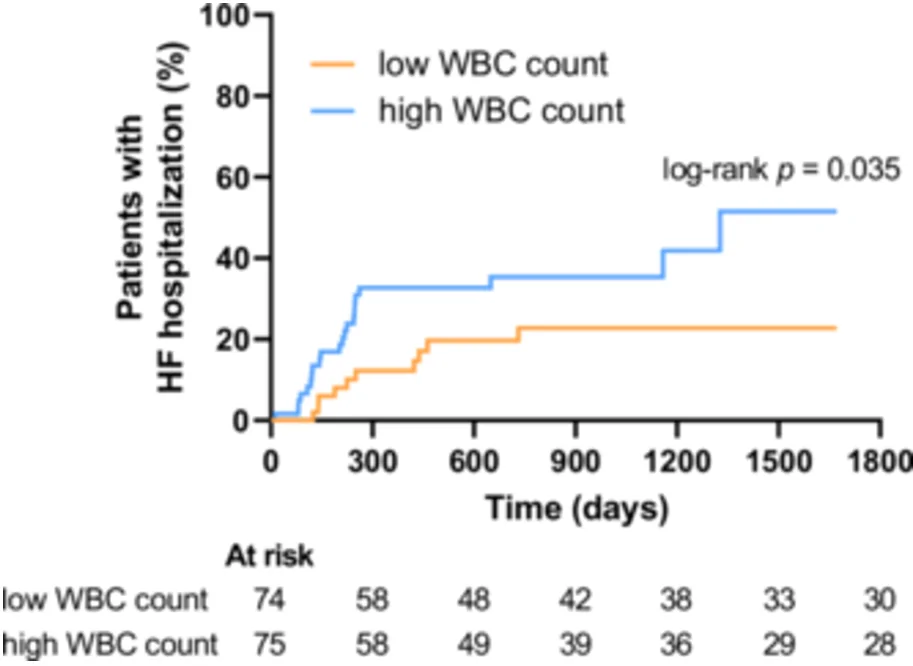
Kaplan−Meier cumulative event curve for hospitalisation due to heart failure with cardiac amyloidosis patients separated by low and high white blood cell (WBC) count.

**Figure 4:**
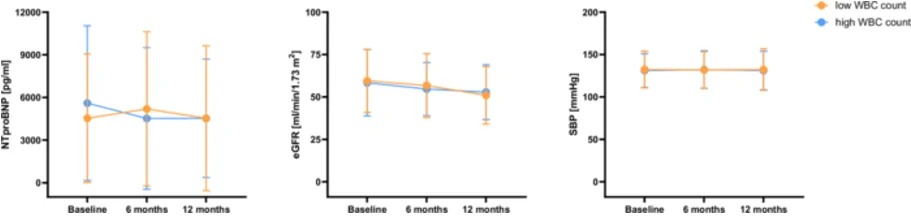
Trend of N‐terminal pro b‐type natriuretic peptide (NT‐proBNP), estimated glomerular filtration rate (eGFR) and systolic blood pressure (SBP) during follow‐up in cardiac amyloidosis patients separated by low and high white blood cell (WBC) count.

**Figure 5:**
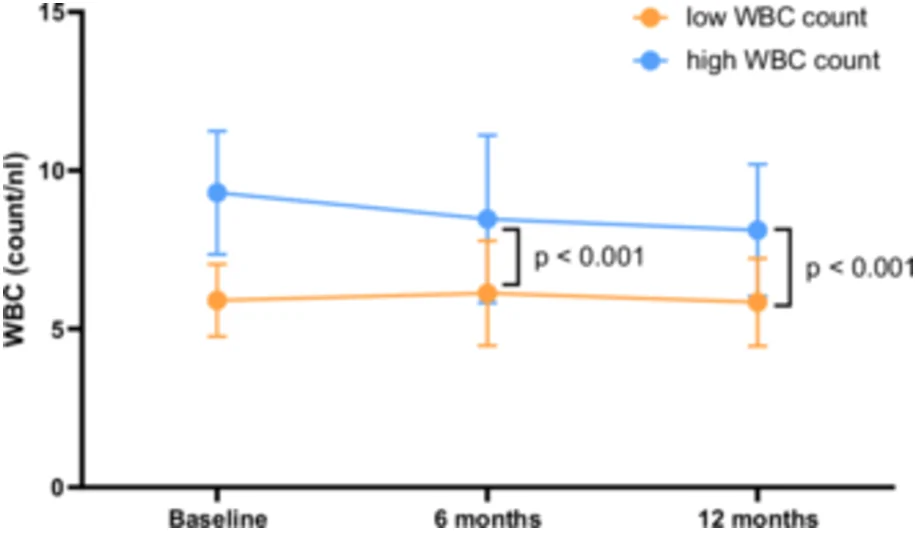
Trend of white blood cell (WBC) count during follow‐up in cardiac amyloidosis patients separated by low and high WBC count at baseline.

## 4 Discussion

This is the first study analysing the association between WBC count and incidence of hospitalisation due to heart failure in ATTR‐CM patients. Patients with higher WBC count showed increased rates of hospitalisation for heart failure, independent from comorbidities, concomitant medication, or established clinical biomarkers. This particularly included cardiovascular diseases, which might provoke heart failure hospitalisation, and heart failure medication influencing disease progression [22, 23].

Endomyocardial biopsy studies revealed the presence of macrophages, lymphocytes and neutrophils in the myocardium of some amyloidosis patients [19, 24]. Detectable myocardial immune cell infiltration is associated with increased risk for all‐cause mortality and hospitalisation due to heart failure [19]. In amyloid light chain (AL) amyloidosis, enhanced WBC count is associated with disease progression in some patients [25]. Similarly, in patients with familial Mediterranean fever (FMF), an inherited autoinflammatory disorder, increased markers of inflammation are associated with the development of amyloid A (AA) amyloidosis [26, 27]. All these previous observations are matching with our findings of increased WBC count being associated with higher rates of hospitalisation due to heart failure in patients with ATTR‐CM. Furthermore, we found an increased prevalence of HFrEF in ATTR‐CM patients with high WBC count, indicating a potential association of higher inflammatory activity with severity of cardiac amyloidosis (Table 5).

**Table 5:** Univariable and multivariable Cox regression for WBC count and hospitalisation for heart failure.

	Estimated hazard ratio	95% confidence interval	*p* value
Univariable Cox regression	1.130	1.015‐1.257	0.025^a^
Multivariable Cox regression			
Model 1 (age, sex)	1.134	1.017‐1.264	0.023^a^
Model 2 (age, sex, comorbidities)	1.203	1.028‐1.407	0.021^a^
Model 3 (baseline medication)	1.204	1.044‐1.389	0.011^a^
Model 4 (clinical biomarkers)	1.142	1.008‐1.295	0.037^a^

*Note:* Model 1: Adjustment for age and sex; Model 2: Adjustment for age, sex, hypertension, dyslipidemia, diabetes mellitus, history of smoking, obesity, coronary artery disease, history of percutaneous coronary intervention or stroke, coronary artery bypass graft, peripheral artery disease, heart failure subgroups, aortic valve stenosis, mitral and tricuspid valve regurgitation, cardiac device, atrial fibrillation and chronic obstructive pulmonary disease; Model 3: Adjustment for heart failure medication, anticoagulation, antiplatelet therapy, statins and disease‐modifying therapy; Model 4: Adjustment for left ventricular ejection fraction, systolic blood pressure, hemoglobin, platelet count, C‐reactive protein (CRP), estimated glomerular filtration rate, high‐sensitive troponin and N‐terminal prohormone of brain natriuretic peptide (NT‐proBNP).

Abbreviation: WBC, white blood cell.

^a^ Statistically significant association of WBC count and hospitalisation for heart failure.

Analysis of immune cell subtypes revealed a relative increase of neutrophils accompanied by a relative decrease of lymphocytes in ATTR‐CM patients with high WBC count in our study. Accordingly, this led to an increase in NLR in patients with increased WBC count. We additionally found enhanced absolute counts of thrombocytes in patients with higher WBC counts, as platelets are activated during enhanced inflammatory response [28, 29]. Although neutrophils do not directly mediate the clearance of systemic amyloid deposits, they can be activated by amyloid and assist in amyloid removal via professional phagocytes such as macrophages [30]. Furthermore, neutrophil proteases were found to be associated with amyloid fibrils in tissue biopsies from amyloidosis patients [31]. Also, neutrophil markers like NLR or serum azurocidin, a neutrophil‐derived granule protein, were associated with development of amyloidosis in FMF patients [32, 33]. As the increase in WBC count in our cohort was driven by a relative increase in neutrophils, association with increased incidence of hospitalisation due to heart failure might be linked to a relevant involvement of neutrophil activity in patients with ATTR‐CM, which needs further investigation in the future.

With increasing development and approval of disease‐modifying drugs, monitoring of therapy response and disease progression will get more important in clinical practice. Therefore, valid biomarkers are needed to support potential therapy adaptation. With upcoming phase 3 clinical trials to assess treatment efficacy of monoclonal antibodies designed to deplete amyloid deposits in ATTR‐CM, monitoring of inflammatory response will also become essential [10]. In this context, WBC count and NLR might serve as potential surrogate markers for amyloid‐induced inflammatory response or biomarkers for therapy efficacy. In our study cohort of ATTR‐CM patients, tafamidis was used as disease‐modifying therapy. As this treatment acts as a transthyretin stabilizer, remaining myocardial amyloid deposits might provoke an ongoing inflammatory response [34, 35, 36]. Accordingly, WBC count remained significantly elevated in ATTR‐CM patients with increased WBC count at baseline in our study, indicating sustained inflammatory activity. In future studies investigating new immune therapies, capable of reducing cardiac amyloidosis in a more effective way in ATTR‐CM patients, inflammatory response and WBC count might be important targets for monitoring therapy efficacy.

Patients with ATTRv amyloidosis represent a special ATTR‐CM subgroup. ATTRv amyloidosis results from various mutations in the TTR gene with geographical differences [37, 38, 39]. ATTRv patients show differences in clinical phenotype, including a variance‐dependent development of polyneuropathy (ATTRv‐PN) and age at disease onset [40, 41]. The pathophysiological impact of inflammation was investigated in several studies of patients with ATTRv‐PN. Here, mutant TTR protein was associated with infiltration of immune cells and production of inflammatory cytokines [42]. Upregulation of several inflammatory plasma markers applicable for monitoring of disease progression and therapy response was observed in ATTRv‐PN patients [43, 44]. The role of inflammation in patients with ATTRv amyloidosis with predominant cardiomyopathy and the impact of different TTR mutations have not been substantially investigated so far. In our study, only seven patients with ATTRv cardiac amyloidosis were identified, which does not allow further valid deductions. With emerging therapeutic options, reliable biomarkers for early diagnosis and screening of family members with a positive genotype will be essential for patients with ATTRv amyloidosis [45, 46]. In this context, inflammatory biomarkers might be promising targets in future studies.

### 4.1 Limitations

This study has several limitations. Our patient collective is rather small and derived only from a single specialized center, which may limit generalizability. In this context, the use of the median split approach provides only limited information on clinically applicable cut‐off values for WBC count. Further studies with larger patient cohorts are required to address this limitation in a more statistically robust manner. CRP was not a relevant confounder in our study. Still, neutrophil granule peptides or cytokines as neutrophil‐specific proinflammatory indicators may have influenced patient outcomes and the underlying pathophysiological mechanisms. Combined with the retrospective design of our study, this does not allow to deduce a causal relationship between WBC count and heart failure hospitalisation. Also, only 13.3% of our patients were female, reflecting the epidemiology of ATTR‐CM, but limiting the translation of our findings to this patient subgroup [47]. As high‐sensitivity CRP (hs‐CRP) is associated with heart failure hospitalisation in multiple HFpEF trials and these study collectives include higher portions of female patients, another limitation of our study is missing information on hs‐CRP [48, 49, 50, 51]. Although adjustments were made for concomitant cardiovascular comorbidities as potential sources of inflammation, data on the prevalence of infections as an additional source of inflammatory activity were not available in this study [52]. To address those limitations, larger prospective multi‐center studies with comprehensive analysis of potential underlying mechanisms are needed to validate our findings in the future. Also, studies focusing on female patients with ATTR‐CM are needed to evaluate appropriate inflammatory biomarkers in this special subgroup.

## 5 Conclusion

This study evaluated the association of WBC count and hospitalisation for heart failure in patients with cardiac amyloidosis. ATTR‐CM patients with higher WBC count showed an increased prevalence of HFrEF. Thereby, higher WBC count was driven by relative increase in neutrophils and relative decrease of lymphocytes, resulting in enhanced NLR. Patients with higher WBC count showed increased incidence of hospitalisation for heart failure. This association remained significant after adjustment for further confounders like comorbidities, concomitant medication and other established clinical biomarkers. These results give new insights in pathophysiological mechanisms for cardiac amyloidosis and might improve risk stratification, diagnostics and therapy in ATTR‐CM patients in the future.

## Author Contributions

Conceptualization: Elias Haj‐Yehia, Julia Vogel and Lars Michel. Methodology: Elias Haj‐Yehia, Julia Vogel, Samuel Dautzenberg, Sophia Jura, Phillip Schulte, Stephan Settelmeier, Florian Buehning, Tobias Lerchner, Loubna Yahsaly, and Lars Michel. Software, not applicable; validation: Elias Haj‐Yehia, Julia Vogel, Samuel Dautzenberg, Phillip Schulte, and Lars Michel. Formal analysis: Elias Haj‐Yehia, Julia Vogel, Samuel Dautzenberg, and Phillip Schulte. Investigation: Elias Haj‐Yehia, Julia Vogel, Samuel Dautzenberg, Sophia Jura, Phillip Schulte, Stephan Settelmeier, Florian Buehning, Tobias Lerchner, Loubna Yahsaly, and Lars Michel. Resources: Elias Haj‐Yehia, Julia Vogel, Alexander Carpinteiro, Christian Reinhardt, Matthias Totzeck, Tienush Rassaf, and Lars Michel. Data curation: Elias Haj‐Yehia, Julia Vogel, and Lars Michel. Writing–original draft preparation: Elias Haj‐Yehia and Julia Vogel. Writing–review and editing: Alexander Carpinteiro, Christian Reinhardt, Matthias Totzeck, Tienush Rassaf, and Lars Michel. Visualization: Elias Haj‐Yehia and Julia Vogel. Supervision: Matthias Totzeck, Tienush Rassaf, and Lars Michel. Project administration: Matthias Totzeck, Tienush Rassaf, and Lars Michel. Funding acquisition: Elias Haj‐Yehia, Julia Vogel, Alexander Carpinteiro, Christian Reinhardt, Matthias Totzeck, Tienush Rassaf, and Lars Michel. All authors have read and agreed to the published version of the manuscript.

## Conflicts of Interest

The authors declare no conflicts of interest.

## Supporting information


**Table S1:** Genetic information and WBC count for patients with hereditary ATTR‐CM (ATTRv).

## Data Availability

The dataset analyzed during the current study is available from the corresponding author upon reasonable request.
