# Supplementary material for: White Blood Cell Count Is Associated With Hospitalisation for Heart Failure in Patients With ATTR Cardiac Amyloidosis
Source: Clin Cardiol. 2026 Nov 10;49(11):e70525. doi: 10.1002/clc.70525 (PMC13750107; doi:10.1002/clc.70525)
Supplement: Supplementary file 1 [file CLC-49-e70525-s001.docx]

**Supplemental Table S1:** Genetic information and WBC count for patients with hereditary ATTR-CM (ATTRv)

| **Patient number** | **Genetic mutation** | **WBC (count/nl)** | | |
| --- | --- | --- | --- | --- |
|  |  | **Baseline** | **6 months** | **12 months** |
| Patient #1 | p.Val122Ile | 4.22 | 4.22 | 4.32 |
| Patient #2 | p.Ser43Asn | 6.22 | 8.43 | 6.65 |
| Patient #3 | p.Arg34Gly | 8.85 | 6.98 | 7.04 |
| Patient #4 | p.Ile88Leu | 7.76 | 11.61 | 10.37 |
| Patient #5 | p.Ala65Thr | 7.86 | 7.13 | 8.72 |
| Patient #6 | p.Leu78His | 11.75 | 6.49 | 6.78 |
| Patient #7 | p.Val122Ile | 7.96 | 8.18 | 6.70 |

WBC, white blood cell; ATTR-CM, transthyretin amyloid cardiomyopathy
